# Cardiolipin remodelling in mitochondrial therapeutics: translational evidence chains from elamipretide to emerging strategies

**DOI:** 10.3389/fphys.2026.1813119

**Published:** 2026-05-29

**Authors:** Ke Di, Yusheng Hu, Hechen Sun, Tianwei Meng, Tingquan Han, Rui Qie

**Affiliations:** 1 Heilongjiang University of Chinese Medicine, Harbin, China; 2 Heilongjiang Provincial Academy of Traditional Chinese Medicine, Harbin, China; 3 Heilongjiang Provincial Hospital of Traditional Chinese Medicine, Harbin, China; 4 First Affiliated Hospital, Heilongjiang University of Chinese Medicine, Harbin, China

**Keywords:** cardiolipin, cardiolipin remodelling, elamipretide, inner mitochondrial membrane, target engagement

## Abstract

Mitochondrial therapeutics have repeatedly fallen short of disease modification, in part because inner-membrane architecture constrains both molecular access and the reversibility of bioenergetic failure. Cardiolipin (CL), the signature phospholipid of the inner mitochondrial membrane, provides a mechanistically cohesive axis that links membrane mechanics to cristae organisation, respiratory-chain supercomplex stability and redox vulnerability. This Review summarises how CL biosynthesis and remodelling shape tissue-specific lipid species and how pathological remodelling can amplify oxidative injury. The MLCL: CL ratio is discussed as a rare lipid biomarker with direct interpretability for diagnosis, stratification and target engagement. Clinical evidence for CL-targeting stabilisers is evaluated to delineate settings in which structural support yields functional benefit and those in which organ-level remodelling limits translation despite improved respiration. Emerging approaches that improve tissue distribution, modulate membrane phase behaviour or rebalance upstream lipid flux are considered alongside trial designs that couple target-proximal readouts to patient-relevant endpoints, paving the way for precision medicine in mitochondrial lipodystrophies.

## 1 Introduction

Mitochondrial medicine has occupied an awkward position in biomedical research: implicated in an extraordinary range of human disorders, yet yielding remarkably few therapies that alter disease course (Zong et al., 2024). From inherited defects of oxidative phosphorylation to cardiometabolic syndromes and neurodegeneration linked to ageing, mitochondrial dysfunction recurs as a mechanistic theme (Gropman et al., 2024). Translation, however, has faced persistent hurdles (Gropman and Chandra, 2024). The problem is not a lack of plausible biology; it is that mitochondria present a set of constraints that challenge conventional drug-development playbooks. The inner mitochondrial membrane (IMM) is one such constraint. It is engineered to sustain a steep electrochemical gradient and to segregate redox chemistry from the cytosol (Li et al., 2024). Molecules that reach this compartment must do so without collapsing membrane potential, perturbing respiratory-chain organisation, or creating off-target liabilities across tissues with very different energetic demands. Many programmes have therefore converged on downstream readouts-oxidative stress, redox balance, generic “bioenergetic support”-that are easy to measure and attractive in principle, but are often decoupled from the structural determinants of mitochondrial performance (Gropman et al., 2024). Clinical heterogeneity compounds the issue. “Mitochondrial disease” is a spectrum of failure modes, not a single entity; pooling nuclear DNA maintenance defects with primary mitochondrial DNA (mtDNA) translation lesions can merge mechanistically incompatible pathologies and flatten therapeutic signals.

A more structural view has emerged, built on advances in cryo-electron microscopy, lipidomics, and membrane biophysics (Jiang et al., 2022). Within this view, cardiolipin (CL) becomes an unusually powerful explanatory and therapeutic axis. CL is the signature phospholipid of the IMM, enriched at sites where curvature, protein crowding, and proton handling are most intense (Domitin et al., 2025). Its distinctive four–acyl chain scaffold supports negative curvature and helps maintain cristae architecture, the geometry that concentrates oxidative phosphorylation machinery and shapes local energetic microenvironments (Makowski et al., 2025). CL also participates directly in lipid–protein interfaces that stabilise respiratory-chain supercomplexes (Yi et al., 2022). When CL is depleted, oxidised, or pathologically remodelled, supercomplex integrity weakens, electron transfer becomes less efficient, and electron leak rises-conditions that amplify reactive oxygen species (ROS) formation and further damage the lipid environment (Jiang et al., 2022). In this framing, CL is not a passive “membrane component” but a keystone that couples membrane mechanics to respiratory efficiency (Goncalves et al., 2021).

Regulatory events in 2025 brought this mechanistic shift into the clinic. The US Food and Drug Administration granted accelerated approval to elamipretide (Forzinity) for improving muscle strength in adults and paediatric patients with Barth syndrome weighing at least 30 kg, anchoring the decision to knee extensor muscle strength as an intermediate endpoint and requiring confirmatory evidence of clinical benefit (Shirley, 2025). It legitimises a strategy in which stabilising the IMM lipid environment can deliver clinically meaningful functional gains even when the causal mutation remains uncorrected. It also signals that, in ultra-rare metabolic disease, an intermediate endpoint anchored in pathophysiology-such as quantified muscle strength-may be accepted as “reasonably likely” to predict clinical benefit, provided the evidentiary chain is coherent and confirmatory studies are pursued. Yet the same therapeutic class has produced divergent outcomes across indications. In primary mitochondrial myopathy, *post-hoc* analyses have suggested genotype-dependent responsiveness, with signals concentrated in subsets defined by nuclear DNA-driven mtDNA maintenance defects and little benefit in primary mtDNA translation disorders. In heart failure, improvements in cellular respiration have not reliably translated into organ-level functional change, raising the possibility that fibrosis, extracellular matrix remodelling, and macrostructural constraints can dominate the phenotype and limit what membrane stabilisation can achieve alone.

This Review examines CL-centred therapeutics through a translational lens. It connects CL biosynthesis and remodelling with membrane biophysics and respiratory supercomplex organisation. It focuses on the balance between Tafazzin-dependent maturation and ALCAT1-associated pathological remodelling. It also highlights newly defined substrate-flux control at the cardiolipin-to-monolysocardiolipin conversion step mediated by ABHD18. Beyond summarising current evidence, this Review advances three linked propositions: first, cardiolipin-directed stabilisers are most likely to yield interpretable benefit when residual respiratory architecture remains biologically rescuable; second, in fibrosis- or macroremodelling-dominant phenotypes, improved mitochondrial respiration may not propagate to organ-level benefit; third, CL-state readouts are valuable not only for diagnosis, but also for stratification, target engagement, and endpoint design (Vaz et al., 2022; Shirley, 2025). It then evaluates the clinical evidence base for elamipretide across Barth syndrome, mitochondrial myopathy, and heart failure, before considering emerging modalities designed to address limitations in tissue distribution, central nervous system access, and phase instability (including SBT-272 and HDAP2) alongside gene-replacement approaches. The goal is therefore not only to review existing studies, but also to define the biological boundary conditions under which CL-targeting strategies are most plausibly disease-modifying and most appropriately tested in future trials.

## 2 Biological basis of cardiolipin-targeted membrane therapies

**Figure 1:**
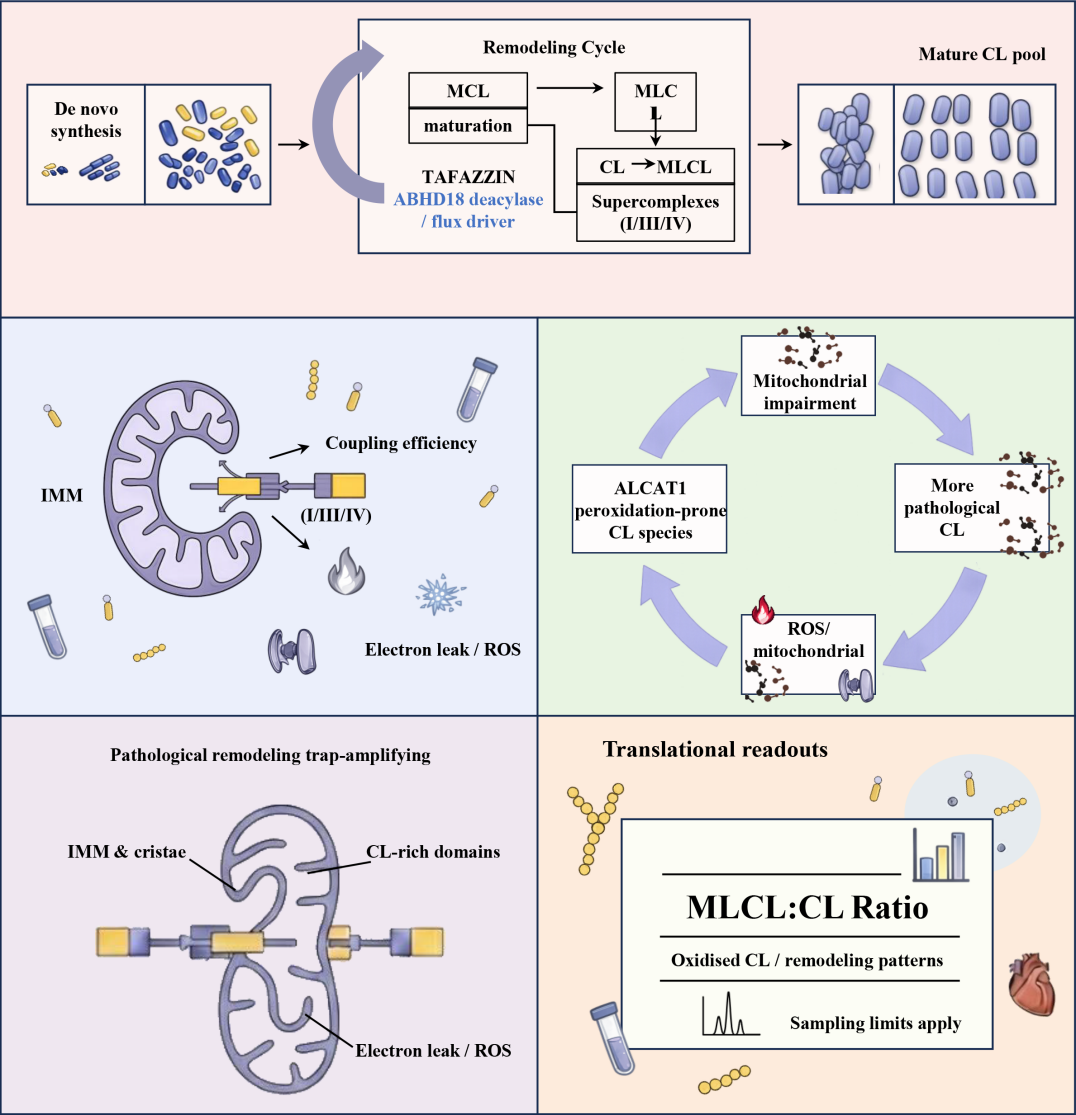
The lifecycle of cardiolipin from homeostatic remodeling to pathological amplification. *De novo* synthesis feeds into a remodeling cycle governed by Tafazzin and the deacylase ABHD18, generating the mature CL species required for respiratory supercomplex stability. Under stress, ALCAT1 drives a maladaptive loop that incorporates oxidation-prone acyl chains, amplifying ROS production. The MLCL: CL ratio serves as a translational biomarker reflecting this remodeling efficiency.

CL occupies an unusual position in mitochondrial biology. It is both a constitutive lipid of the IMM and an active determinant of how respiratory proteins assemble, communicate, and fail under stress. Any therapeutic strategy framed as membrane stabilisation is best judged against the biochemical reality of CL’s lifecycle, including how CL is synthesised, how it is remodelled into tissue-specific species, and how maladaptive remodelling under stress creates a lipid environment that amplifies oxidative injury rather than resisting it (**Figure 1**).

### 2.1 CL shapes cristae and supercomplex order

CL is distinguished from canonical glycerophospholipids by its dimeric architecture. Two phosphatidyl moieties bridged by a glycerol backbone yield four acyl chains and a compact headgroup. This geometry favours negative curvature and supports the high-curvature topology of cristae, where oxidative phosphorylation is physically concentrated (Spikes et al., 2020; Hryc et al., 2023). Cristae are increasingly viewed as microcompartments in which proton handling, protein crowding, and diffusion constraints co-determine bioenergetic output (Domitin et al., 2025). It is also worth retaining a bidirectional view of proton dynamics at the CL-rich inner membrane: under physiological conditions, the anionic headgroup can act as a local “proton trap”, increasing surface proton affinity and supporting microdomain supply; yet when CL is oxidised, depleted, or spatially disordered, the same interfacial chemistry may plausibly shift toward non-productive surface conduction and increased non-specific proton leak, eroding the ability to sustain membrane potential. This framing suggests that membrane stabilisers such as elamipretide may help not only by preserving supramolecular organisation but also by improving the physical properties of pathological CL domains in ways that indirectly reduce futile leak and improve coupling efficiency-without implying a single mandatory mechanism (Prola et al., 2021). Within this setting, CL contributes to supramolecular organisation of the electron transport chain, stabilising higher-order assemblies often termed supercomplexes or respirasomes (Senoo et al., 2020; Corey et al., 2022; Hryc et al., 2023). When CL content, acyl composition, or oxidation state shifts, the penalty is rarely confined to a single enzyme. The architecture that supports efficient electron flow becomes mechanically and electrostatically less coherent, with predictable consequences for electron leak and reactive oxygen species (ROS) formation.

### 2.2 Tafazzin remodelling and the MLCL: CL signature

Newly synthesised CL is not born fit for purpose. *De novo* pathways generate CL species whose acyl chains reflect substrate availability rather than organ-specific optimisation, leaving an immature and heterogeneous pool (Oemer et al., 2020; Chen et al., 2021). Functional maturation requires remodelling through a deacylation–reacylation cycle that produces tissue-enriched species with characteristic symmetry and packing behaviour. Tafazzin, encoded by TAZ, is central to this process. Tafazzin operates as a transacylase, transferring acyl chains between phospholipids and monolysocardiolipin (MLCL), shaping a mature CL spectrum aligned with organ demands (Abe et al., 2016; Anzmann et al., 2021). In heart and skeletal muscle, the mature profile is enriched in tetralinoleoyl CL, a species associated with tight packing and support of high-flux oxidative metabolism (Le et al., 2020).

The diagnostic and mechanistic signature of impaired tafazzin activity is not simply low CL. A more discriminating marker is the MLCL: CL ratio, which captures a bottleneck at the remodelling step. MLCL accumulates, mature CL is depleted, and the membrane inherits a detergent-like liability that destabilises bilayer order (Vaz et al., 2022). The clinical utility of MLCL: CL as a biomarker in Barth syndrome is widely recognized (Thompson et al., 2016) and repeatedly shown to outperform absolute CL content for diagnostic discrimination (Byeon et al., 2021).

### 2.3 ALCAT1 and the self-amplifying lipid trap

Tafazzin-dependent remodelling supports biochemical fitness. In acquired metabolic disease, CL remodelling can become maladaptive. ALCAT1 has been implicated as a driver of pathological CL remodelling under oxidative stress and nutrient excess (Song et al., 2019; Jia et al., 2021). Mechanistic studies associate ALCAT1 activity with incorporation of highly unsaturated acyl chains into CL, producing species vulnerable to peroxidation. The consequence is not merely oxidative damage downstream of mitochondrial dysfunction. It can become a self-reinforcing loop in which remodelled CL species facilitate further ROS production, mitochondrial impairment, and metabolic derangement (Hao et al., 2024). Genetic deletion or suppression of ALCAT1 has been reported to mitigate aspects of mitochondrial dysfunction and metabolic pathology in relevant models, supporting a role upstream of oxidative vulnerability rather than as a passive bystander (Liu et al., 2022; Wang et al., 2025).

This distinction matters for membrane-targeted therapeutics. A peptide or small molecule that binds CL may shield existing lipid domains from physical disintegration, yet it does not automatically neutralise an enzymatic programme that continually repopulates the membrane with oxidation-prone species. Under such conditions, structural stabilisation is forced into competition with ongoing biochemical rewiring of lipid composition. Stabilisers may therefore perform best when the membrane retains a sufficiently coherent substrate pool, even if quantitatively reduced, whereas enzymatically driven remodelling can render the target unstable in a dynamic, self-renewing fashion (Jia et al., 2021; Hao et al., 2024).

### 2.4 ABHD18 flux control and the bypass logic

Beyond “physical stabilisation” and “restoring tafazzin function,” a 2025 *Nature* study proposed a third therapeutic logic for Barth syndrome. It is a bypass-oriented strategy. The key is to suppress ABHD18. This aims to reduce pathological substrate flux through the cardiolipin-to-monolysocardiolipin deacylation step (Masud et al., 2025). ABHD18 was characterised as a CL deacylase capable of converting CL into MLCL, positioning it as a candidate enzymatic source of MLCL accumulation when tafazzin-dependent remodelling is impaired (Ren et al., 2025). In patient-derived cells and animal models of tafazzin deficiency, ABHD18 inactivation reduced abnormal MLCL build-up, improved mitochondrial ultrastructure and function, and ameliorated disease-relevant outcomes; the same work reported leads consistent with selective covalent small-molecule inhibition that partially rescues tafazzin-mutant phenotypes in patient systems.

Crucially, the therapeutic intent is neither to “stabilise the membrane” in the presence of ongoing MLCL production nor to “repair the causal gene”, but to lower MLCL generation pressure so that residual or compensatory remodelling capacity can re-establish a viable steady state. This substrate-flux strategy generates directly testable translational endpoints, including MLCL: CL ratio, nascent CL abundance and acyl composition, respiratory-chain supercomplex stability, and functional readouts such as muscle performance and cardiac phenotypes in relevant models.

### 2.5 Biomarkers that travel from bench to trial

The MLCL: CL ratio is a rare lipid biomarker that remains both mechanistically interpretable and clinically actionable (Xu et al., 2022). In tafazzin deficiency, MLCL accumulation is not merely a correlative signal: it reports a structural liability in the IMM with implications for cristae organisation, supercomplex stability and cytochrome release (Díaz-Quintana et al., 2020; Vaz et al., 2022; Hryc et al., 2023). In acquired cardiometabolic disease, the informative axis may shift from absolute abundance toward CL quality-including oxidation state, acyl symmetry and remodelling flux-where the same ratio can be less binary yet still conceptually anchored to membrane vulnerability (Jia et al., 2021).

For translation, the practical task is to define lipid readouts that can serve two roles simultaneously: confirming target engagement and separating mechanistic subtypes that are likely to respond to membrane-directed intervention. MLCL: CL provides a robust anchor in inherited disease, while broader lipidomic fingerprints-such as oxidised CL species or remodelling patterns consistent with ALCAT1-associated flux-may become more decisive in acquired settings, where inflammation and redox pressure co-evolve with lipid turnover (Jia et al., 2021; Masud et al., 2025).

Despite its specificity, MLCL: CL remains constrained by sampling logistics for longitudinal follow-up and pharmacodynamic inference. Measurement has historically relied on specialised mass-spectrometric workflows in patient cells or blood-derived fractions, and even more accessible matrices still face limitations in resolving organ- or compartment-level engagement (Byeon et al., 2021; Bautista et al., 2022). A pragmatic response is to develop repeatable “liquid-biopsy” surrogates, including extracellular vesicles enriched for mitochondrial cargo and mitochondria-derived vesicles, alongside mitochondrial microRNA signatures (Amari and Germain, 2021; König and McBride, 2024). These approaches are valuable less for novelty than for interpretability: they help distinguish insufficient exposure from true biological boundaries when tissue sampling is infeasible (D'Acunzo et al., 2021; Picca et al., 2023; Zhou et al., 2023; Hazan Ben-Menachem et al., 2024; Iorio et al., 2024; Kim et al., 2024). Taken together, these considerations suggest that the most useful biomarkers in this field are not necessarily those that are merely disease-associated, but those that can bridge mechanism, patient selection, and longitudinal pharmacodynamic interpretation across indications. In this sense, CL-state readouts should be viewed as part of trial architecture rather than as ancillary biochemical descriptors alone (Bautista et al., 2022; Vaz et al., 2022).

## 3 Elamipretide in the clinic and what it really tests

**Figure 2:**
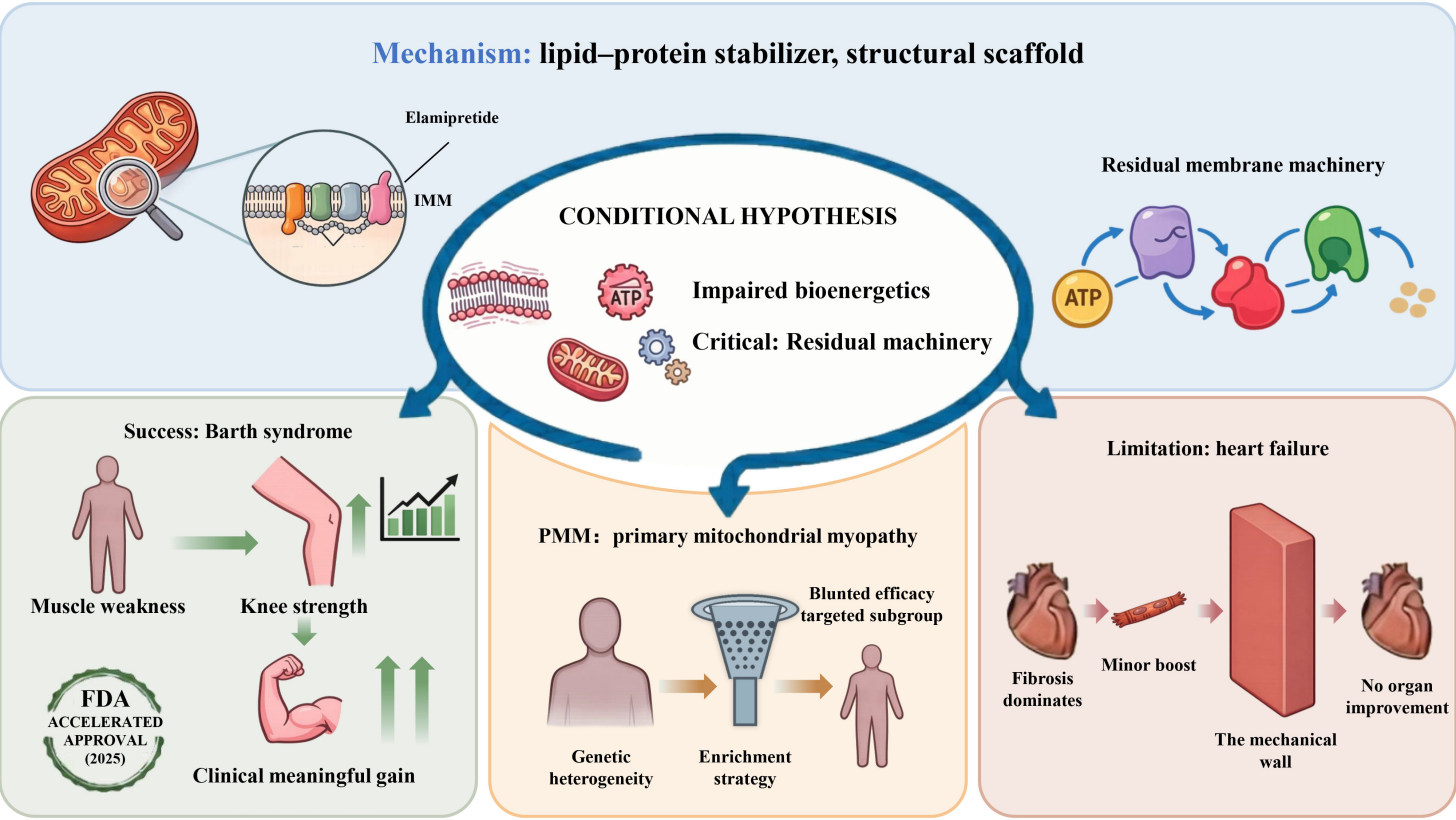
The conditional hypothesis of membrane stabilization across disease contexts. Elamipretide acts as a structural scaffold at the inner mitochondrial membrane, but its efficacy is contingent on residual bioenergetic machinery. In Barth syndrome, this stabilization translates into clinical gains such as improved muscle strength, supporting the 2025 FDA approval. Conversely, in heart failure, macroscopic fibrosis acts as a “mechanical wall” that decouples cellular bioenergetic improvements from organ-level functional recovery. In Primary Mitochondrial Myopathy, genetic heterogeneity necessitates enrichment strategies to identify responsive subgroups.

Elamipretide’s clinical record is best interpreted as a test of a membrane-architecture hypothesis rather than a direct repair of cardiolipin remodelling. Because the intervention acts at the lipid–protein interface, its probability of success depends on residual respiratory-chain machinery, the proximity of chosen endpoints to mitochondrial mechanics, and the duration required for structural stabilisation to manifest as measurable function. The following subsections synthesise what different trial settings reveal about these boundary conditions, from accelerated approval in Barth syndrome to the dilutional effects of heterogeneity and the limits imposed by fibrosis-dominant heart failure (**Figure 2**).

### 3.1 Stabilising structure without repairing biochemistry

**Figure 3:**
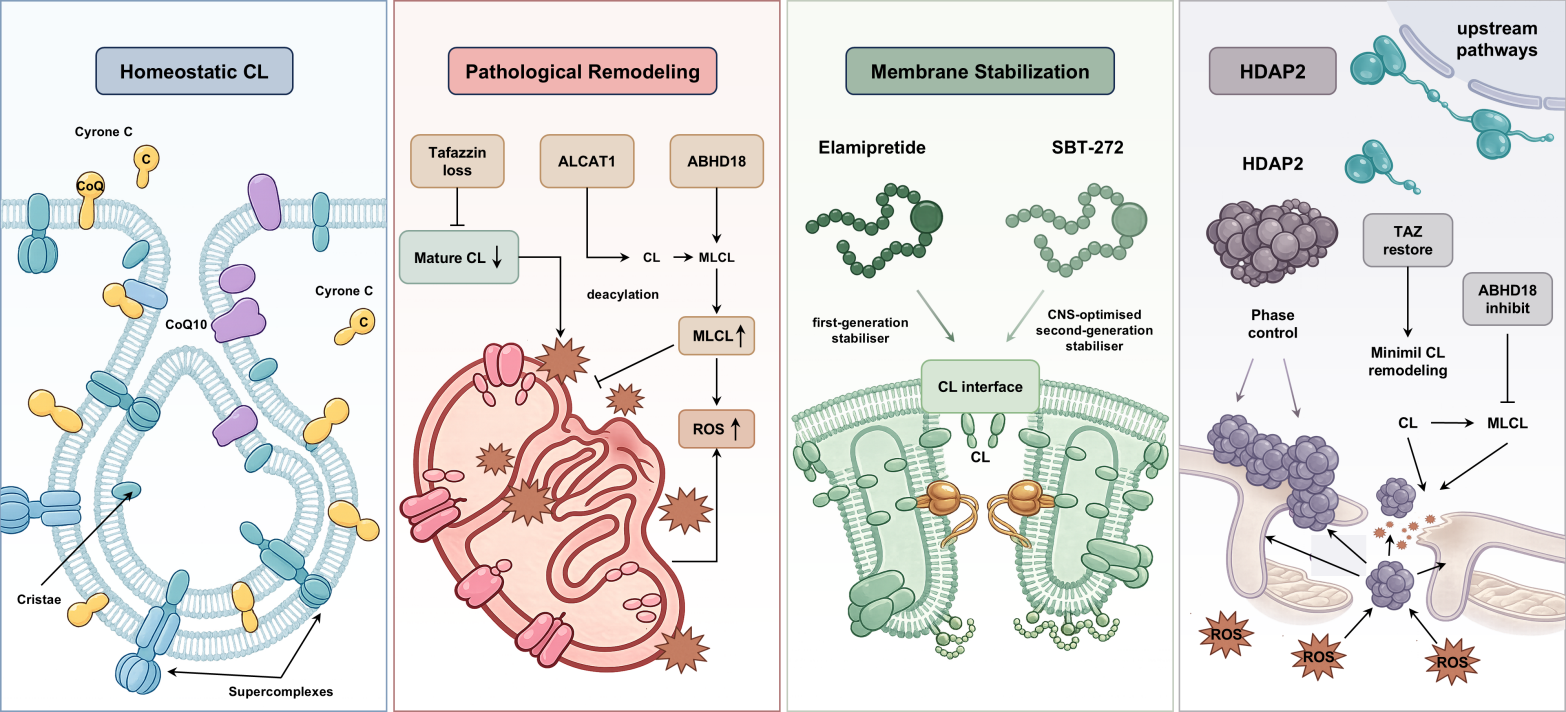
Distinct mechanistic logics of cardiolipin-targeted therapeutics at the inner mitochondrial membrane. Elamipretide and SBT-272 are illustrated as cardiolipin-directed membrane stabilizers that act at CL-rich inner mitochondrial membrane interfaces to preserve cristae architecture, support respiratory-chain organization, and improve coupling efficiency. HDAP2 is shown as a phase-modulating strategy designed to preserve membrane state under severe stress through supramolecular assembly. For mechanistic contrast, upstream remodeling and flux-control pathways involving Tafazzin, ALCAT1, and ABHD18 are also indicated.

Elamipretide is best understood as a pharmacological intervention in membrane architecture (Chavez et al., 2020). Biophysically, it is a mitochondria-targeting aromatic–cationic peptide that partitions into cardiolipin-enriched inner mitochondrial membrane domains rather than acting through a conventional receptor-mediated mechanism. Current evidence suggests that, after electrostatic attraction to anionic phospholipids, elamipretide associates with CL-containing membrane interfaces, modulates membrane surface electrostatics, and helps preserve local lipid packing and cristae curvature, thereby supporting the supramolecular organisation of oxidative phosphorylation complexes. In this setting, its downstream effects are proposed to include improved coupling efficiency, reduced non-productive proton leak, attenuation of ROS amplification, and stabilisation of respiratory-chain supercomplex-associated membrane architecture, especially when a recoverable bioenergetic apparatus is still present (Sabbah et al., 2025). Importantly, elamipretide does not restore Tafazzin-dependent cardiolipin remodelling or directly normalise the MLCL: CL ratio; rather, it functionally supports the residual CL pool and the membrane environment built upon it. This places elamipretide more precisely in the category of cardiolipin-directed membrane stabilisation than in that of biochemical repair of cardiolipin remodelling (Tung et al., 2025) (**Figure 3**).

### 3.2 Barth syndrome and the accelerated-approval blueprint

Forzinity received accelerated approval in the United States in 2025 for improving muscle strength in adults and paediatric patients with Barth syndrome weighing at least 30 kg. The approval is tied to knee extensor muscle strength as an intermediate endpoint, with continued approval contingent on a postmarketing confirmatory trial. The prescribing information specifies once-daily subcutaneous dosing (40 mg) for eligible patients and classifies the drug as a mitochondrial CL binder. The evidentiary package cites a controlled period followed by an open-label extension in which functional strength signals became clearer over longer exposure, a pattern consistent with a membrane-architecture intervention whose macroscopic readouts accrue gradually (Hornby et al., 2022; Thompson et al., 2024). Beyond the approval itself, this decision is important because it illustrates current regulatory expectations in ultra-rare mitochondrial diseases. The FDA’s acceptance of a strength-based intermediate endpoint, explicitly tied to a mechanistically coherent chain from inner membrane biology to functional performance, offers a template for future programmes in which conventional hard endpoints are impractical yet pathophysiological linkage can be demonstrated with sufficient rigour (Allen et al., 2020; Sabbah et al., 2025).

### 3.3 Primary mitochondrial myopathy and the challenge of heterogeneity

The development narrative in primary mitochondrial myopathy has been more equivocal, reflecting the constraints of broad inclusion criteria in a genetically diverse spectrum disorder. MMPOWER-3 illustrates how genetic heterogeneity can blunt efficacy signals when endpoints are dominated by whole-body performance measures and the underlying defect varies from “assembly inefficiency” to “loss of essential components” (Karaa et al., 2023). Trial registration records, together with subsequent interpretative literature, situate MMPOWER-3 within that methodological context.

NuPOWER represents a strategic pivot toward enrichment, narrowing eligibility to subgroups more likely to retain sufficient residual machinery for membrane-level optimisation to translate into measurable function. The trial registration documentation reflects this move toward genotype aligned design. For a membrane stabiliser, the practical implication is straightforward: the more intact the remaining bioenergetic apparatus, the greater the probability that improved lipid–protein organisation can yield clinically meaningful gains (Flickinger et al., 2021; Gwaltney et al., 2022; Montano et al., 2022).

### 3.4 Heart failure and the wall of macroscopic remodelling

Experience in acquired heart failure has clarified the limits of a membrane-first strategy. In PROGRESS-HF, short-term intravenous elamipretide did not yield clear improvement in left-ventricular structural endpoints such as left ventricular end-systolic volume, despite a rationale grounded in cellular energetics (Butler et al., 2020). For heart failure with preserved ejection fraction (HFpEF), trial registry records are available, but public reporting of definitive clinical efficacy remains limited; RESTORE-HF (NCT02814097) is listed as completed, yet outcomes have not been consistently disseminated in peer-reviewed form. That gap is now complemented by more concrete mechanistic evidence from a recent 2026 preclinical study in female obese ZSF1 rats, a cardiometabolic HFpEF model in which elamipretide produced a modest increase in complex I/II-linked mitochondrial respiration without translating into improvement in diastolic performance or mechanical indices, while hypertrophy- and fibrosis-linked remodelling readouts remained dominant (Schauer et al., 2026). This pattern supports a stage-structured interpretation in metabolic HFpEF: mitochondrial inefficiency can function as a contributor or amplifier, but once extracellular matrix remodelling and macrostructural stiffness are established, improving respiratory-chain efficiency alone is unlikely to overcome the constraints imposed by fibrosis and reduced organ-level compliance (Paulus and Zile, 2021; Borlaug et al., 2023; Weil et al., 2025). The broader inference for trialists is that bioenergetic rescue at the cellular level may be necessary yet insufficient once macroscopic remodelling, fibrosis, or geometric distortion dominates the phenotype. In such settings, time of intervention, combinatorial regimens, and biomarker anchored stratification become central design variables rather than optional refinements.

### 3.5 Chronic dosing realities and safety signals

The prescribing information specifies a clear dosing framework, weight threshold, and renal impairment adjustments in adults, along with warnings related to benzyl alcohol exposure and hypersensitivity reactions. Injection site reactions constitute the most common adverse events, consistent with long-term subcutaneous peptide administration (Thompson et al., 2024). These attributes collectively support the feasibility of chronic management in Barth syndrome, while highlighting constraints that will shape any broader expansion, including treatment burden and the evidential demands of extrapolation beyond a tightly defined rare-disease population (**Table 1**).

**Table 1:** Human evidence for elamipretide across major clinical indications.

Indication	Key study/programme	Study design	Main endpoint(s)	Main finding	Reader-friendly takeaway	Key source(s)
Barth syndrome	TAZPOWER + OLE	Randomized, double-blind, placebo-controlled crossover trial with open-label extension	6-minute walk distance; muscle strength; patient-reported outcomes	Functional benefit became clearer with longer treatment.	Strongest human support is in Barth syndrome.	PMID: 33077895
PMM	MMPOWER	Early randomized signal-finding study	6-minute walk distance	An early exercise-related efficacy signal was observed.	Early study supported proof-of-concept in PMM.	PMID: 29500292
PMM	MMPOWER-2	Phase 2 randomized crossover trial	6-minute walk test; fatigue-related outcomes	Mid-stage results suggested benefit but were not confirmatory.	Signal was encouraging, but evidence remained preliminary.	PMID: 32096613
PMM	MMPOWER-3	Phase 3 randomized placebo-controlled trial	6-minute walk distance; PMMSA fatigue score	The trial did not meet its co-primary endpoints.	Unselected PMM may be too heterogeneous for a clear signal.	PMID: 37268435
Nuclear DNA-defined primary mitochondrial disease	NuPOWER	Enriched phase 3 randomized trial	Functional and fatigue-related outcomes	The study is ongoing in a genetically selected population.	Tests whether biological enrichment improves response detection.	ClinicalTrials.gov NCT05162768
HFrEF	PROGRESS-HF	Phase 2 randomized placebo-controlled trial	LV end-systolic volume; LV ejection fraction	No clear short-term improvement in LV structure was seen.	Mitochondrial benefit may not translate into organ-level gain.	PMID: 32068002
HFpEF	RESTORE-HF	Phase 2 randomized placebo-controlled trial	Imaging-based LV function; exercise-related endpoints	Trial completion is known, but efficacy results remain unclear.	Clinical evidence in HFpEF is still incomplete.	ClinicalTrials.gov NCT02814097

OLE, open-label extension; PMM, primary mitochondrial myopathy; PMMSA, Primary Mitochondrial Myopathy Symptom Assessment; HFrEF, heart failure with reduced ejection fraction; HFpEF, heart failure with preserved ejection fraction; LV, left ventricular.

## 4 Second waves of CL targeting

CL–targeted therapeutics are consolidating into a “membrane-to-function” paradigm: interventions intended to reshape the inner-mitochondrial-membrane microenvironment and lipid–protein organisation, strengthen respiratory-chain architecture, and improve stress resilience with a mechanistically interpretable route to functional outcomes. This section focuses on two representative strategies. Exposure- and distribution-led redesign aims to make CL-centric mechanisms testable in central nervous system (CNS) indications despite blood–brain barrier constraints. Phase-behaviour control frames CL targeting as membrane engineering, using programmable supramolecular organisation to stabilise membrane state beyond single-site binding. The section closes by distilling the evidentiary hierarchy, falsifiable readouts, and endpoint-mapping rules required for translation-ready programmes.

### 4.1 SBT-272 and the CNS-access problem

A recurring limitation of first-generation CL-targeting peptides is not the plausibility of the target but the practical reach of the modality. A strategy that can be evaluated in heart and skeletal muscle becomes difficult to interrogate in neurodegeneration when blood–brain barrier permeability constrains exposure and compresses the window for mechanistic validation. SBT-272 (bevemipretide) can be understood as a second-generation cardiolipin-targeting peptide designed to retain the membrane-centred logic of elamipretide while improving tissue distribution to the CNS. Mechanistically, it is proposed to act at the CL-rich inner mitochondrial membrane interface, where it helps preserve mitochondrial ultrastructure, maintain membrane potential, and support mitochondrial transport under stress, rather than correcting upstream cardiolipin remodelling enzymes or replacing deficient lipids *de novo*. In this sense, the distinguishing feature of SBT-272 is not a fundamentally different molecular target, but improved CNS exposure that makes cardiolipin-directed membrane stabilisation experimentally and translationally more accessible in neurodegenerative settings (**Figure 3**).

Its appeal in amyotrophic lateral sclerosis is tied to a relatively concrete mitochondrial interface. TAR DNA-binding protein 43–linked pathology can be framed as a proximal mitochondrial insult with downstream effects on motility and axonal integrity (Carroll et al., 2026); in corticospinal motor neuron systems carrying mutant TAR DNA-binding protein 43, SBT-272 has been reported to improve mitochondrial motility and ultrastructure, with axonal outgrowth used as a functional readout (Genin and Paquis-Flucklinger, 2026). Early clinical progress has been communicated through sponsor-facing disclosures, including phase 1 safety signals and orphan drug designation, which together support feasibility but do not yet establish efficacy (Gautam et al., 2023).

Taken together, the current evidence for SBT-272 remains predominantly preclinical, with early human safety context but no established clinical efficacy. Accordingly, SBT-272 is best framed as a CNS-oriented extension of cardiolipin-directed membrane stabilisation, with the key translational question being whether improved exposure can convert target-proximal mitochondrial effects into durable patient-relevant benefit (Cummings et al., 2025).

### 4.2 HDAP2 and phase control as membrane engineering

HDAP2 represents a different escalation of the membrane-stabilisation paradigm. Rather than functioning primarily as a small cardiolipin-binding peptide at a discrete membrane interface, high-density aromatic peptides are proposed to self-assemble on damaged mitochondrial membranes and to regulate local phase behaviour in CL-rich domains. In biochemical terms, this strategy aims to prevent stress-induced non-bilayer transitions, preserve membrane state and membrane potential, and reduce oxidative amplification under conditions in which simple electrostatic stabilisation may be insufficient (Birk et al., 2025; MacNeil et al., 2026). HDAP2 should therefore be viewed less as a direct analogue of elamipretide and more as a supramolecular membrane-engineering approach within the broader cardiolipin-targeted field (Posey et al., 2023; Balakrishnan and Kenworthy, 2024; Ikari et al., 2024) (**Figure 3**).

The optic nerve injury setting provides a relatively interpretable testbed for this concept. In an optic nerve crush model, daily treatment has been linked to improved retinal ganglion cell survival, with electron microscopy indicating marked attenuation of mitochondrial loss within injured axons and partial to near-restoration of mitochondrial density under daily treatment (Alberti et al., 2025; MacNeil et al., 2026), consistent with a mechanism framed as structural asset preservation under extreme stress. These observations speak most directly to structural asset preservation under severe stress, which is precisely the domain in which phase locking is hypothesised to outperform simple electrostatic stabilisation.

The same features that make HDAP2 mechanistically distinctive create translational uncertainty. Micrometre-scale assemblies raise questions about distribution, clearance, and the relationship between local engagement and systemic exposure. At present, HDAP2 mainly illustrates a phase-control strategy within the broader cardiolipin-targeted field, but major translational questions remain unresolved (Ikari et al., 2024).

### 4.3 Shared rules for translation-ready programmes

Across CNS-penetrant stabilisers and phase-locking assemblies, the discipline is converging on a harder requirement than generic bioenergetic readouts. Target engagement must be demonstrable in metrics that sit close to membrane architecture and respiratory chain organisation, and these metrics must map to functional endpoints with reproducible directionality (Hermans et al., 2025; Jeng and Siegel, 2025). That is the boundary between a compelling mechanistic narrative and a programme that can sustain clinical scrutiny (Seibyl, 2023).

## 5 Comparative framework of stabilisation, phase control, repair, and flux strategies

**Figure 4:**
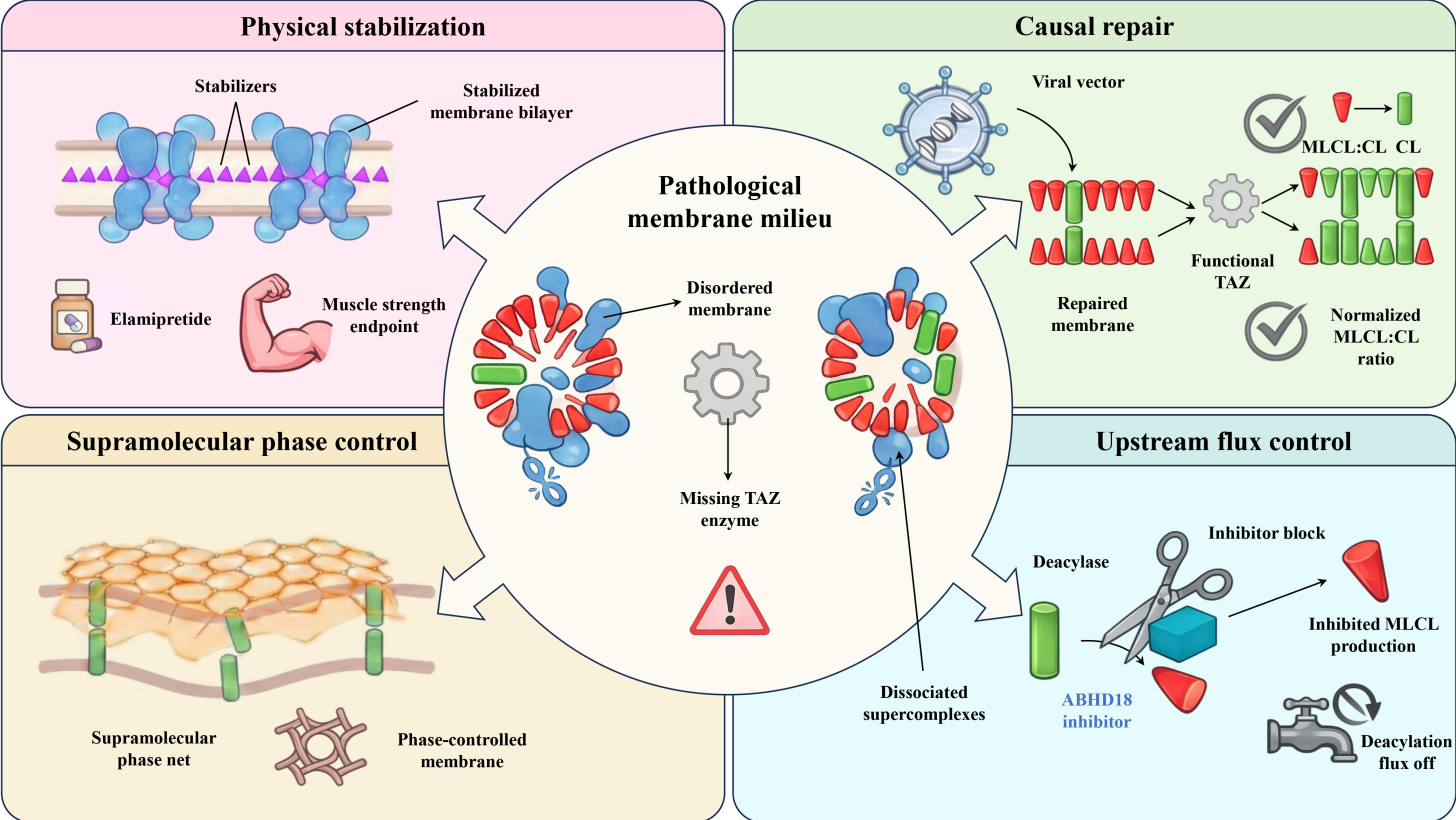
A diversified portfolio of cardiolipin-targeted therapeutic strategies. The current landscape encompasses four distinct paradigms: physical stabilization of the lipid bilayer, such as Elamipretide; causal repair via gene therapy, such as AAV-TAZ, to restore remodeling capacity; supramolecular phase control using phase-modulating peptides, exemplified by HDAP2, to prevent non-bilayer transitions; and upstream flux control targeting deacylases, including ABHD18 inhibition, to limit monolysocardiolipin accumulation.

The field has moved beyond proof-of-concept toward a comparative evaluation of multiple therapeutic strategies (**Figure 4**). The decisive questions now concern controllability of a damaged inner-membrane milieu, predictability of tissue exposure, and whether a given modality can sustain a coherent chain from target engagement to clinically interpretable function (Fuentes and Morcillo, 2024). Strategically, current programmes can be understood as four parallel paradigms: physical stabilisation of the inner mitochondrial membrane (elamipretide-class agents); supramolecular phase control via higher-order assemblies (HDAP2-like constructs); causal restoration of cardiolipin remodelling capacity through adeno-associated virus–mediated TAZ gene replacement; and upstream control of substrate flux that functionally bypasses tafazzin by limiting cardiolipin deacylation to monolysocardiolipin through ABHD18 inhibition (Birk et al., 2025). The regulatory anchor is the accelerated approval of Forzinity (elamipretide) for Barth syndrome in adults and paediatric patients weighing at least 30 kg, granted on the basis of improvement in knee extensor muscle strength as an intermediate clinical endpoint, with confirmatory requirements embedded in the label and approval documentation (Shirley, 2025). This framework sets an implicit comparator: subsequent approaches must either outperform the first-generation stabiliser on exposure and mechanistic readouts at the same target, or shift upstream to causal repair of remodelling, accepting a different risk–benefit profile (**Table 2**) (Fuentes and Morcillo, 2024).

**Table 2:** Comparative framework of cardiolipin-targeted therapeutic strategies.

Strategy class	Representative example	Mechanistic level	What it aims to correct	Evidence status	Major strength	Major limitation	Main indication focus	Key source(s)
Physical stabilisation	Elamipretide (Forzinity)	CL-directed membrane stabilisation at the inner mitochondrial membrane	Destabilised CL-rich membrane microenvironment	Approved for Barth syndrome	Has the strongest human and regulatory support in the field	May be insufficient once organ-level remodelling dominates	Barth syndrome; PMM; heart failure	PMID:33077895
Physical stabilisation	SBT-272 (bevemipretide)	CNS-oriented CL-directed membrane stabilisation	Same CL-centred membrane problem, with added CNS exposure limitation	Preclinical/early clinical development	Extends the stabilisation concept toward brain-penetrant indications	Clinical efficacy remains unproven	ALS and other neurodegenerative disorders with mitochondrial involvement	PMID:36716828
Supramolecular phase control	HDAP2	Phase control/supramolecular membrane engineering	Stress-induced membrane phase instability	Preclinical	Mechanistically distinct from simple CL binding; designed for severe membrane stress states	Biodistribution and clearance remain uncertain	Optic nerve injury/retinal neurodegeneration	PMID:41035613
Causal repair	AAV-TAZ gene replacement	Gene restoration of tafazzin function	Primary tafazzin deficiency/defective CL maturation	Preclinical	Addresses the upstream genetic cause rather than compensating downstream	Delivery, immunogenicity, and irreversible vector exposure	Barth syndrome	PMID:31336787
Upstream flux control	ABHD18 inhibition	Substrate-flux control of CL-to-MLCL deacylation	Excess CL-to-MLCL flux and MLCL accumulation	Discovery/preclinical	Mechanistically precise way to reduce pathological MLCL generation pressure	Translational feasibility is still being defined	Barth syndrome/tafazzin-deficient systems	PMID:40903572

CL, cardiolipin; MLCL, monolysocardiolipin; PMM, primary mitochondrial myopathy; ALS, amyotrophic lateral sclerosis; CNS, central nervous system.

### 5.1 Membrane stabilisation and distribution limits

Forzinity anchors the stabilisation paradigm as a regulatorily legible model of CL-directed therapy: a chronic, peripherally delivered intervention explicitly framed as a mitochondrial CL binder. The core wager is architectural rather than biochemical (Mitchell et al., 2020). Elamipretide does not restore CL remodelling capacity; it aims to stabilise an existing CL pool and improve lipid–protein coherence such that respiratory assemblies can operate more efficiently (Allen et al., 2020). The corollary is conditional but clear. Benefit is most plausible where impaired performance is driven by a destabilised membrane microenvironment and a disorganised lipid–protein interface that remains, at least in part, recoverable; benefit is harder to translate where organ-level endpoints are dominated by entrenched macroscopic remodelling, extracellular matrix constraint, or fibrosis-driven stiffness that cannot be reversed by improved respiration alone.

Second-wave programmes that retain the CL-stabilisation logic increasingly treat exposure as a first-order design variable rather than a downstream optimisation. In practice, the same mechanistic narrative becomes far less interrogable when the relevant tissue is inaccessible and when negative outcomes can reflect insufficient engagement as much as insufficient biology (Gautam et al., 2023). This exposure constraint is especially decisive for neuro-mitochondrial indications, where blood–brain barrier penetration determines whether target-proximal endpoints can be linked to functional readouts. SBT-272 (bevemipretide) is best read as an exposure-led redesign that preserves the CL logic while pursuing sustained CNS levels after systemic dosing, thereby turning previously hard-to-test mechanistic bridges into tractable experimental chains.

Within this framework, stabilisation is most likely to yield interpretable benefit when the residual bioenergetic apparatus remains sufficiently intact and when disease stage has not progressed beyond a structurally reversible phase (Allen et al., 2020). Conversely, stabilisation becomes intrinsically ambiguous when exposure is uncertain or when the phenotype is dominated by non-mitochondrial structural constraints, because improved respiration can be real yet clinically silent. The practical implication for programme design is therefore not simply “choose better endpoints”, but to pair architecture-proximal engagement measures with indication-appropriate functional readouts in contexts where exposure and rescuable biology can be demonstrated rather than assumed (Fuentes and Morcillo, 2024).

### 5.2 Phase regulation and mechanistic repair

A distinct escalation in the CL portfolio shifts competition from “binding a lipid” toward “engineering a phase landscape”. HDAP2 exemplifies this vector by proposing that CL-rich membranes fail not only through loss of organisation but also through stress-driven transitions into non-bilayer or otherwise catastrophic states, and that controlling phase behaviour can preserve membrane potential and structural assets when simple stabilisation is insufficient (Birk et al., 2025). The conceptual novelty is matched by translational uncertainty. Micrometre-scale assemblies raise questions about biodistribution, clearance, and how local engagement relates to systemic exposure; accordingly, phase-control programmes are best judged by falsifiable, architecture-proximal endpoints that report phase behaviour, ultrastructure preservation and coupling efficiency, rather than by generic redox readouts alone.

Orthogonal strategies move upstream from membrane physics to causal capacity and substrate pressure. Gene replacement of TAZ aims to restore Tafazzin function and normalise the MLCL: CL signature (Wang et al., 2020), thereby addressing the remodelling bottleneck rather than compensating for its consequences (Suzuki-Hatano et al., 2019). The translational bargain differs from chronic stabilisation: potential durability and causal correction are traded against systemic delivery requirements across heart and skeletal muscle, immunological constraints, dose ceilings, and the irreversibility of adeno-associated virus exposure. Disease stage remains a major effect modifier in a way that must be stated plainly-late fibrosis and architectural collapse may not be reclaimed even if remodelling capacity is restored-making early-window intervention or tightly defined subpopulations the most defensible development path (Zhu et al., 2021).

Between stabilisation and gene repair lies a bypass paradigm. It targets lipid flux rather than membrane structure. ABHD18 suppression has been proposed to reduce pathological cardiolipin deacylation and thereby limit monolysocardiolipin formation. This may lower MLCL accumulation and allow residual or compensatory remodelling to re-establish a viable steady state in tafazzin-deficient systems (Ren et al., 2025). This logic preserves the translational emphasis of the CL field: it yields directly testable endpoints including MLCL: CL ratio, nascent CL abundance and acyl composition, supercomplex stability and functional readouts in relevant models. More broadly, it clarifies why stabilisation can break in acquired disease contexts. When an enzymatic remodelling programme and oxidative pressure continuously repopulate the membrane with vulnerable species, a purely physical stabiliser may be forced into competition with a dynamically renewing pathological lipid environment; under such conditions, durable benefit is more plausibly achieved by upstream repair, flux control, or rational combinations that jointly address membrane stability and lipid turnover.

### 5.3 Reading the evidence matrix without overreach

Forzinity’s approval rests on the TAZPOWER evidence package and the regulatory acceptance of knee extensor muscle strength as an intermediate clinical endpoint (Shirley, 2025). More importantly, this approval highlights the need to link inner-membrane engagement and structural readouts to a clinically meaningful functional outcome in rare disease settings (Reid Thompson et al., 2021).

MMPOWER-3 supplies the counterpoint. When genetically and mechanistically distinct primary mitochondrial myopathy populations are aggregated, conventional functional endpoints are prone to signal dilution (Karaa et al., 2023). *Post-hoc* analyses reported a more coherent response signal in nuclear DNA–related subgroups, motivating NuPOWER’s enrichment design aimed at isolating patients whose residual respiratory apparatus may be salvageable by membrane stabilisation. NuPOWER therefore examines whether genetic stratification can improve signal detection for a membrane-stabilising therapy in a biologically selected population (Karaa et al., 2024).

Heart failure studies function most usefully as boundary conditions. Clinical investigations such as PROGRESS-HF underline that improvements in mitochondrial respiration do not automatically translate into left-ventricular structural or haemodynamic endpoints over short time horizons (Butler et al., 2020). HFpEF-oriented preclinical work further implies that a metabolically driven, inflammatory lipid milieu may persist, limiting the capacity of physical stabilisation alone to reprogramme upstream remodelling. Within the evidence matrix, these data refine indications, timing, and the rationale for combination approaches rather than negate the membrane-stabilisation concept. .

## 6 Key translational questions for future development

The limiting challenge for CL stabilisers is not whether mitochondrial respiration can be improved, but whether such improvement can propagate into durable, interpretable clinical benefit across disease contexts. In Barth syndrome, functional signals tend to accrue with extended exposure, whereas in heart failure populations bioenergetic optimisation is frequently constrained by fibrosis, geometry, and other macroscopic mechanical determinants. In cardiometabolic settings, persistent oxidative stress and pathological lipid remodelling can also reshape cardiolipin faster than a purely physical stabiliser can hold the system, undermining durability at the lipidomic level. This section focuses on three determinants: timing and the boundary of reversibility, the conditions under which remodelling defeats stabilisation, and the operational definition of “rescuable substrate” for precision enrolment and endpoint selection (**Table 3**).

**Table 3:** Candidate translational readouts for cardiolipin-targeted therapies.

Readout level	Example readouts	What it informs	Most relevant strategy classes	Key source(s)
Target-proximal	MLCL: CL ratio; CL acyl composition/individual CL species	Whether the intervention directly changes the CL state or lipid remodeling signature	Causal repair; upstream flux control; physical stabilisation	PMID: 34382226 PMID: 34273557
Mechanism-proximal	Respiratory-chain supercomplex organisation; cristae ultrastructure; mitochondrial membrane potential; coupled respiration	Whether membrane architecture and bioenergetic mechanism are actually improved	Physical stabilisation; supramolecular phase control; causal repair	PMID: 37188665 PMID: 33523852
Functional/tissue-level	Knee extensor muscle strength; 6-minute walk distance; axonal transport/axonal outgrowth; cardiac imaging endpoints	Whether mitochondrial improvement propagates to tissue- or organ-level function	Physical stabilisation; CNS-oriented membrane stabilisation; supramolecular phase control	PMID: 33077895 PMID: 36716828 PMID: 32068002
Patient-relevant	Exercise capacity; fatigue; symptom burden; disease progression	Whether patients experience meaningful and clinically interpretable benefit	All strategy classes, especially those entering clinical translation	PMID: 38602181 PMID: 33077895 PMID: 38602181

Examples are representative rather than exhaustive. Readout selection should depend on indication, disease stage, and mechanism of action. In inherited CL-remodeling defects, target-proximal readouts are especially useful for confirming lipid-state correction; in membrane-stabilising or phase-control strategies, mechanism-proximal readouts are particularly important for linking membrane rescue to downstream functional outcomes. Representative supporting sources for the readout hierarchy and examples listed above include studies on blood-spot MLCL: CL assays, high-resolution CL profiling, CL-dependent supercomplex organisation, CL-dependent coupling efficiency, SBT-272 readouts in TDP-43 models, PROGRESS-HF imaging endpoints, and FDA-recognized muscle-strength endpoints in Barth syndrome.

### 6.1 When timing becomes the main determinant

The clinical narrative around CL stabilisers is dominated by a striking asymmetry. In Barth syndrome, functional benefit appears to accrue over extended exposure (Thompson et al., 2024), whereas in heart failure populations improvements in cellular bioenergetics have not translated reliably into macroscopic haemodynamic or remodelling endpoints (Butler et al., 2020). The FDA’s accelerated approval of Forzinity frames this tension in regulatory terms, accepting knee extensor muscle strength as an intermediate endpoint while requiring confirmatory evidence of clinical benefit (Zhao et al., 2026). This structure implicitly recognises that functional readouts may precede harder outcomes, yet it also forces the field to specify when a bioenergetic intervention can still be “received” by a tissue-level mechanical system.

Current evidence supports the possibility that the cardiac effect of CL stabilisation may decline as remodelling becomes more advanced, with fibrosis burden and extracellular matrix cross-linking serving as potential operational markers of irreversibility (Helali et al., 2025). Accordingly, trials enrolling predominantly late-stage phenotypes may reflect a ceiling imposed by tissue mechanics rather than pharmacology (Neff and Bradshaw, 2021). A credible validation framework would stratify by imaging-defined fibrosis and ventricular geometry, pairing conventional endpoints with mechanistic readouts that can locate the breakpoint in translation (Ghazal et al., 2025).

### 6.2 When lipid remodelling defeats stabilisation

In primary Tafazzin deficiency, the dominant problem is failure to generate mature CL species alongside MLCL accumulation, and a stabiliser can plausibly compensate by occupying and supporting a destabilised lipid–protein interface (Vaz et al., 2022). The regulatory dossier classifies elamipretide as a CL binder and anchors the approval to a mechanistic chain that begins at the inner membrane. Acquired cardiometabolic disease introduces a different adversary. Persistent pathological remodelling and peroxidation can continuously reshape CL, shifting the system faster than a purely physical stabiliser can hold it (Jiang et al., 2022). ALCAT1 has been repeatedly implicated in abnormal CL remodelling, and preclinical studies of ALCAT1 suppression or inhibition have reported mitigation of mitochondrial dysfunction and redox vulnerability in relevant models, supporting the view that pathological remodelling can sit upstream of oxidative amplification (Zhang et al., 2025). A testable proposition is therefore plausible and clinically actionable. In metabolically stressed myocardium, CL stabilisation alone may improve respiration without durably normalising the lipidomic trajectory, whereas combined ALCAT1 inhibition and stabilisation should yield synergistic shifts in CL oxidation patterns, supercomplex integrity and coupled respiration (Jia et al., 2021). This hypothesis is readily addressable with longitudinal lipidomics and structural bioenergetic endpoints, rather than relying on single time-point functional measures (Jia and Shi, 2026).

### 6.3 Defining rescuable biology for precision enrolment

The MMPOWER-3 randomised trial concluded that elamipretide did not improve 6-minute walk test distance or fatigue at 24 weeks in an unselected primary mitochondrial myopathy population. That result does not resolve the mechanism; it highlights heterogeneity (Karaa et al., 2023). NuPOWER was designed to enrich for disease associated with nuclear DNA mutations, explicitly narrowing the target population to reduce mechanistic dilution. In parallel, genotype-specific *post hoc* analyses of MMPOWER-3 have entered the public domain, suggesting signals consistent with dose responsiveness in selected subgroups (Karaa et al., 2024).

These data argue that future enrolment should be based less on disease labels alone and more on evidence of rescuable respiratory organisation. Operationally, such biology may be approximated through integrated assessment of genotype, respiratory-complex assembly, supercomplex abundance, and CL-state fingerprints, allowing membrane stabilisers to be tested in patients whose mechanistic substrate is preserved but disordered rather than irreversibly lost (Karaa et al., 2023; Karaa et al., 2024). The regulatory logic used for accelerated approval provides a template for linking these mechanistic markers to functional intermediates, while confirmatory trials can close the loop to patient-centred outcomes (Zhao et al., 2026).

## 7 Conclusions and outlook

CL-centred mitochondrial therapeutics are most coherent when judged as interventions in inner-membrane architecture rather than as generic “bioenergetic support” (Dudek, 2017; Jiang et al., 2022). The available evidence therefore supports a conditional translational framework rather than a universal therapeutic claim: cardiolipin-directed stabilisation appears most plausible when membrane disorganisation remains mechanistically linked to dysfunction and when tissue-level remodelling has not yet fixed the phenotype. In this context, CL-state fingerprints, including MLCL: CL and related lipidomic features, are valuable not only as biomarkers but also as tools for patient selection, target engagement, and endpoint layering (Butler et al., 2020; Vaz et al., 2022). Future progress will depend less on broader application of the same mechanism than on better alignment among exposure, disease stage, and biologically rescuable substrate.
